# Feasibility and Clinical Acceptability of Automation-Assisted 3D Conformal Radiotherapy Planning for Patients With Cervical Cancer in a Resource-Constrained Setting

**DOI:** 10.1200/GO.23.00050

**Published:** 2023-09-19

**Authors:** Sikudhani Muya, Jerry Ndumbalo, Sarah Kutika Nyagabona, Shaid Yusuf, Dong Joo Rhee, Beatrice Paul Mushi, Benjamin Li, Li Zhang, Surbhi Grover, Mary Feng, I-Chow Hsu, Elia Mmbaga, Katherine Van Loon, Laurence Court, Melody Xu

**Affiliations:** ^1^ Muhimbili University of Health and Allied Sciences, Dar es Salaam, Tanzania; ^2^ Ocean Road Cancer Institute, Dar es Salaam, Tanzania; ^3^ MD Anderson Cancer Center, Houston, TX; ^4^ University of California, San Francisco, Helen Diller Family Comprehensive Cancer Center, San Francisco, CA; ^5^ University of Pennsylvania, Philadelphia, PA; ^6^ University of Oslo, Oslo, Norway; ^7^ Kaiser Permanente Santa Clara, Santa Clara, CA

## Abstract

**PURPOSE:**

The Ocean Road Cancer Institute (ORCI) in Tanzania began offering 3D conformal radiation therapy (3DCRT) in 2018. Steep learning curves, high patient volume, and a limited workforce resulted in long radiation therapy (RT) planning workflows. We aimed to establish the feasibility of implementing an automation-assisted cervical cancer 3DCRT planning system.

**MATERIALS AND METHODS:**

We performed chart abstractions on 30 patients with cervical cancer treated with 3DCRT at ORCI. The Radiation Planning Assistant (RPA) generated a new automated set of contours and plans on the basis of anonymized computed tomography images. Each were assessed for edit time requirements, dose-volume safety metrics, and clinical acceptability by two ORCI physician investigators. Dice similarity coefficient (DSC) agreement analysis was conducted between original and new contour sets.

**RESULTS:**

The average time to manually develop treatment plans was 7 days. Applying RPA, automated same-day contours and plans were developed for 29 of 30 patients (97%). Of the 29 evaluable contours, all were approved with <2 minutes of edit time. Agreement between clinical and RPA contours was highest for the rectum (median DSC, 0.72) and bladder (DSC, 0.90). Agreement was lower with the primary tumor clinical target volume (CTVp; DSC, 0.69) and elective nodal clinical target volume (CTVn; DSC, 0.63). All RPA plans were approved with <4 minutes of edit time. RPA target coverage was excellent, covering the CTVp with median V45 Gy 100% and CTVn with median V45 Gy 99.9%.

**CONCLUSION:**

Automation-assisted 3DCRT contouring yielded high levels of agreement for normal structures. The RPA met all planning safety metrics and sustained high levels of clinical acceptability with minimal edit times. This tool offers the potential to significantly decrease RT planning timelines while maintaining high-quality RT delivery in resource-constrained settings.

## Teaser

Automation may enable high-quality cervical cancer radiation on shorter treatment planning timelines.

## INTRODUCTION

Cancer incidence and mortality is rapidly increasing worldwide, affecting more than 18 million patients per year.^1^ Striking health disparities in cancer care access and outcomes exist between low-middle–income countries (LMICs) and developed countries. LMICs account for 70% of global cancer deaths, millions of which arise in the setting of inadequate prevention or screening and are incurable because of advanced stage at presentation and limitations in human, financial, and material resources to provide life-saving cancer treatment.^1,2^ Improving cancer care capacity is a critical priority. With radiation therapy (RT) indicated in 50%-70% of cancer cases in LMICs, availability of high-quality, efficient RT is critical for global cancer control.^3-6^

**CONTEXT**

**Key Objective**
The study aimed to assess the feasibility of implementing an automation-assisted 3D conformal radiation therapy (3DCRT) planning system for patients with cervical cancer in a resource-constrained setting.
**Knowledge Generated**
The automation-assisted 3DCRT contouring system demonstrated high agreement for normal structures, meeting safety metrics and maintaining clinical acceptability with minimal edit times. It significantly reduced radiation therapy (RT) planning timelines, generating same-day contours and plans for 97% of patients.
**Relevance**
The study’s findings effectively convey the potential benefits and implications of implementing the automation-assisted 3DCRT planning system in resource-constrained settings can expedite RT planning, ensuring timely treatment delivery for cervical cancer patients. The system’s ability to achieve high-quality RT plans with minimal manual editing has the potential to enhance patient access to efficient and effective treatment, addressing the challenges associated with high patient volumes and limited workforce in such settings. This technology holds promise for improving RT workflow efficiency and enhancing treatment outcomes in resource-limited contexts.

In LMICs that have acquired modern RT linear accelerators, scaling up clinical care capacity is an urgent concern. Two major barriers to patient throughput are the time required for physicians to delineate targets and organs at risk (OAR) and the time required for physicists or dosimetrists to generate RT plans. Yet, physicians, physicists, and dosimetrists are in short supply within LMICs.^3^ Full transition to modern RT necessitates increased patient throughput using high-quality, standardized, and scalable strategies.

Digital technology offers a powerful solution to increase patient throughput in LMICs offering 3D conformal radiation therapy (3DCRT). Research in machine learning has already led to the development of several validated algorithms for automated contouring and planning for head and neck, prostate, lung, and other cancers.^7-15^ Although these are mostly intended for developed regions, the Radiation Planning Assistant (RPA) is exclusively designed for implementation in LMICs.^13,14^ The feasibility of using the RPA system to automate bony-anatomy–based 3DCRT plans for cervical cancer was previously demonstrated in a single institution in South Africa, concluding acceptability of automated plans by the treating physicians.^13^

Further work is needed to improve the accessibility, affordability, and timeliness of RT care delivery. To this end, we hypothesize that using the RPA system in resource-constrained settings can result not only in acceptable automated plans, but that it can also introduce a shortened, minimally resource-intensive workflow for cervical cancer 3DCRT treatment planning. In this study, we sought to assess the potential workflow improvements and clinical acceptability of the RPA system for treatment of patients with cervical cancer in Tanzania.

## MATERIALS AND METHODS

### Study Setting

Ocean Road Cancer Institute (ORCI) is a national referral hospital in Dar es Salaam, Tanzania, and is one of the few hospitals in Tanzania offering comprehensive cancer services, including RT and chemotherapy. RT is provided to public patients at a subsidized cost and at cost for insured patients. At ORCI, cervical cancer represents over one third of the patient volume, amounting to >1,500 patients per year.^16^ Nearly all patients with cervical cancer require RT for curative or palliative treatment.

### Intervention

The RPA is a machine-learning–based automated contouring and RT planning tool developed at the MD Anderson Cancer Center and designed for implementation in LMICs.^9,13^ The RPA has the ability to perform bony-landmark–based or soft-tissue–based 3DCRT planning on the basis of clinician preference. The RPA is currently in its Research Use Only phase and not yet available for clinical use. In this study, deidentified computed tomography (CT) simulation images were submitted to the online RPA portal and automated contours and plans are generated within minutes. Automated contours included clinical target volume (CTV) primary, CTV nodal, rectum, bladder, bowel bag, femur_R, and femur_L. Automated RPA soft tissue–based plans were created using autoexpansion of the CTV primary (internal target volume [ITV] derived as an expansion of 10-mm anterior, posterior, superior, and inferior, and 5 mm lateral from CTV and planning target volume [PTV] derived as a uniform 7-mm margin from ITV). The elective nodal CTV coverage encompassed the obturator, presacral, internal iliac, external iliac, and common iliac nodal regions. The RPA 3DCRT whole pelvis planning required physician-led determination of patient suitability for its predefined whole pelvis fields and was not intended for patients with involved LN. The RPA normalization was user-defined. At the request of ORCI clinicians, the automated RPA plans were normalized to PTV V95% = 99% with a requirement for D0.03cc <110%.

### Study Design

We performed a retrospective application of RPA contours and plans to an independent cohort of patients with cervical cancer treated at ORCI between 2019 and 2020. The study was approved by the institutional review boards of Muhimbili University of Health and Allied Sciences, the National Institute of Medical Research of Tanzania, and the University of California, San Francisco.

### Patient Characteristics

We identified 30 consecutive patients through treatment logbooks who received definitive whole-pelvis 3DCRT for cervical cancer from 2019 to 2020 (without the use of RPA). Chart abstraction was performed to collect demographic and clinicopathologic features of their disease, symptoms at presentation, and treatment course details. Patients were staged using the International Federation of Gynecology and Obstetrics 2009 staging system.^17^ Because of lack of routine cross-sectional staging imaging used in this resource-constrained setting, all patients were treated with whole-pelvis 3DCRT as long as abdominal ultrasound and chest x-rays confirmed absence of metastatic or nodal disease.

### RPA Contour and Plan Review

Each RPA contour and plan was downloaded from the web platform and uploaded into ORCI's local treatment planning system for review. Two independent ORCI clinicians (S.M. and J.N.) reviewed each contour and plan. The target and OAR contours were assessed for acceptability according to standard clinical practice at ORCI (accurately encompassing the organs or tumor of interest and including elective coverage of the correct nodal regions with at least 7-mm margin). Plans were assessed according to standard clinical practice at ORCI with ideal PTV V95% >95%, D0.03cc <110%, and Dmax outside the bowel. Edit time was calculated using a stopwatch to measure the time (seconds) required for contour or plan editing before approval. Dice similarity coefficient (DSC) was used to compare RPA and ORCI contour agreement for the structures elective nodal clinical target volume (CTVn), primary tumor clinical target volume (CTVp), rectum, and bladder. Both the RPA and ORCI plans were used to evaluate the volume receiving 45 Gy (V45 Gy) for RPA target contours (RPA-CTVp and RPA-CTVn) and ORCI clinical contours (ORCI-CTVp and ORCI-CTVn) and described using median and IQR. As nonsystematic variations in PTV labeling, summations, and expansions precluded direct comparisons, only CTV contours were compared in this analysis.

### Statistical Analysis

Patient characteristics, RPA contour approval time, RPA plan approval time, and DSC were described using frequency statistics and median and IQR where applicable. Wilcoxon signed rank testing was used to compare contour agreement between ORCI and RPA contours.

## RESULTS

### Patient Characteristics and Baseline Workflows

Characteristics of patient cases selected for study are summarized in Table 1. All patients were symptomatic at the time of diagnosis and had Eastern Cooperative Oncology Group (ECOG) performance statuses (PSs) of 1-2. The most common symptom at diagnosis was vaginal bleeding (97%) followed by lower abdominal pain (67%). The median time from diagnosis to initiation of 3DCRT was 48 days (IQR, 27.5-118.5 days; Table 2). This time exceeded 100 days in nine patients.

**TABLE 1:**
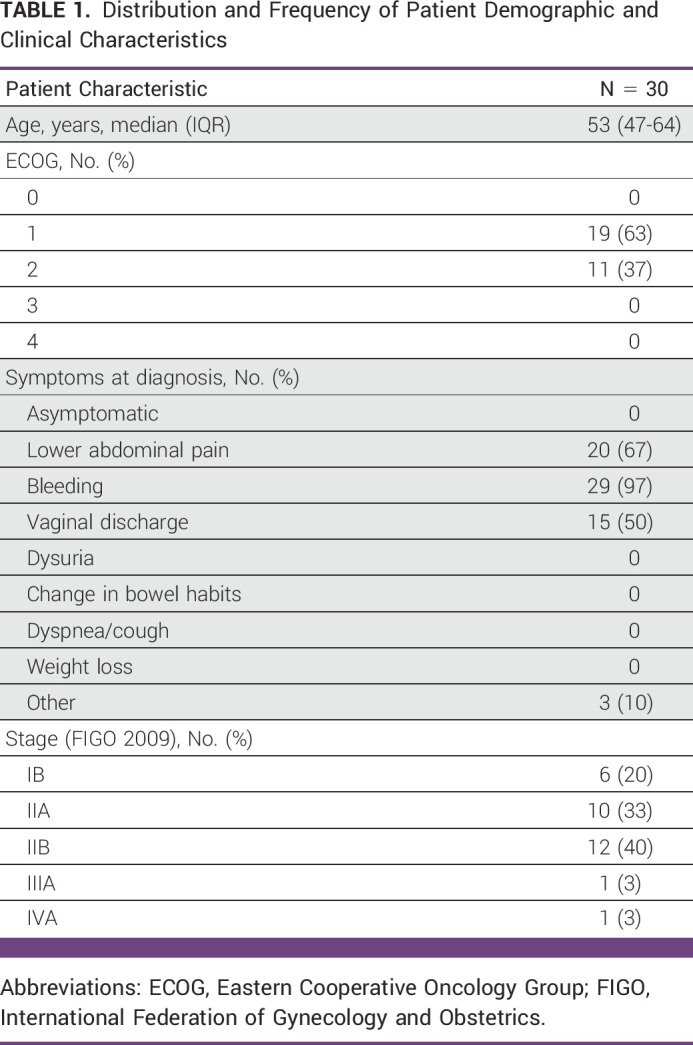
Distribution and Frequency of Patient Demographic and Clinical Characteristics

**TABLE 2:**
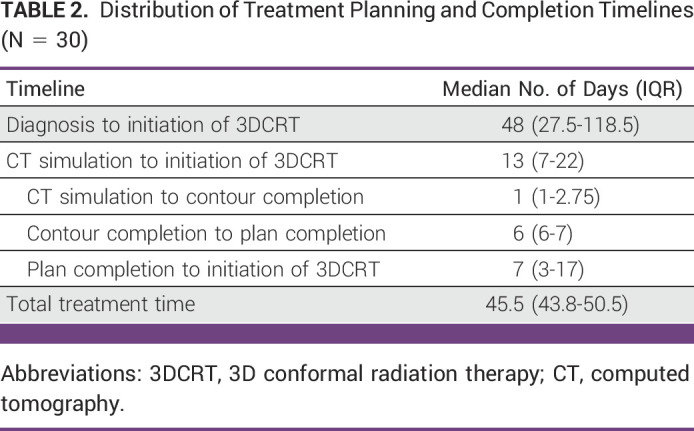
Distribution of Treatment Planning and Completion Timelines (N = 30)

The median time from CT simulation to contour completion was 1 day (IQR, 1-2.75 days) and the median time from contour completion to plan completion was 6 days (IQR, 6-7 days). The median time from plan completion to initiation of 3DCRT was 7 days (IQR, 3-17 days).

The median total treatment time was 45.5 days (IQR, 43.8-50.5 days). Concurrent chemotherapy was administered in 25 patients (83%) and the median number of chemotherapy cycles, given once weekly, was 3 (IQR, 2-4). The most common causes of omission of chemotherapy were elevated creatinine, white blood cell count <2 × 10^9^/L, and hemoglobin <6 g/dL. Brachytherapy was delivered in three implants at 8 Gy per treatment, totaling 24 Gy.

### Time Evaluating Automated Contours and Clinical Acceptability

Using patients' retrospective CT simulation scans, the RPA successfully generated contours and plans for 29 of 30 cases (97%). One patient's CT scan was exported with a single missing slice and therefore RPA contours and plans could not be generated. Of the 29 evaluable contours, all 29 (100%) of contours were approved with an edit time of <120 seconds. The median edit time required for RPA contour approval was 70 seconds (IQR, 64-82 seconds) for the first reviewer and 67 seconds (IQR, 59-71 seconds) for the second reviewer.

**FIG 1:**
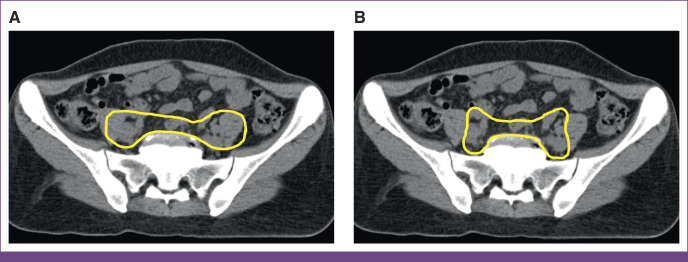
Comparison of pelvic lymph node CTV contours between (A) ORCI and (B) RPA. CTV, clinical target volume; ORCI, Ocean Road Cancer Institute; RPA, Radiation Planning Assistant.

Agreement between clinical and RPA contours was highest for the rectum (median DSC, 0.72; IQR, 0.64-0.77) and bladder (median DSC, 0.90; IQR, 0.88-0.94). Agreement was slightly lower with CTVp (median DSC, 0.69; IQR, 0.58-0.74) and CTVn (median DSC, 0.63; IQR, 0.56-0.72). The RPA contours tended to include more posterolateral margin on vessels, include more presacral nodes, consistently exclude muscle, and included additional extent of CTVp inferiorly (Fig 1).

### Time Evaluating Automated Plans and Clinical Acceptability

Of the 29 plans evaluable, all 29 (100%) were approved with an edit time of <240 seconds. The median edit time required for RPA plan approval was 125 seconds (IQR, 119-133 seconds) for the first reviewer and 146 seconds (IQR, 134-177 seconds) for the second reviewer.

**FIG 2:**
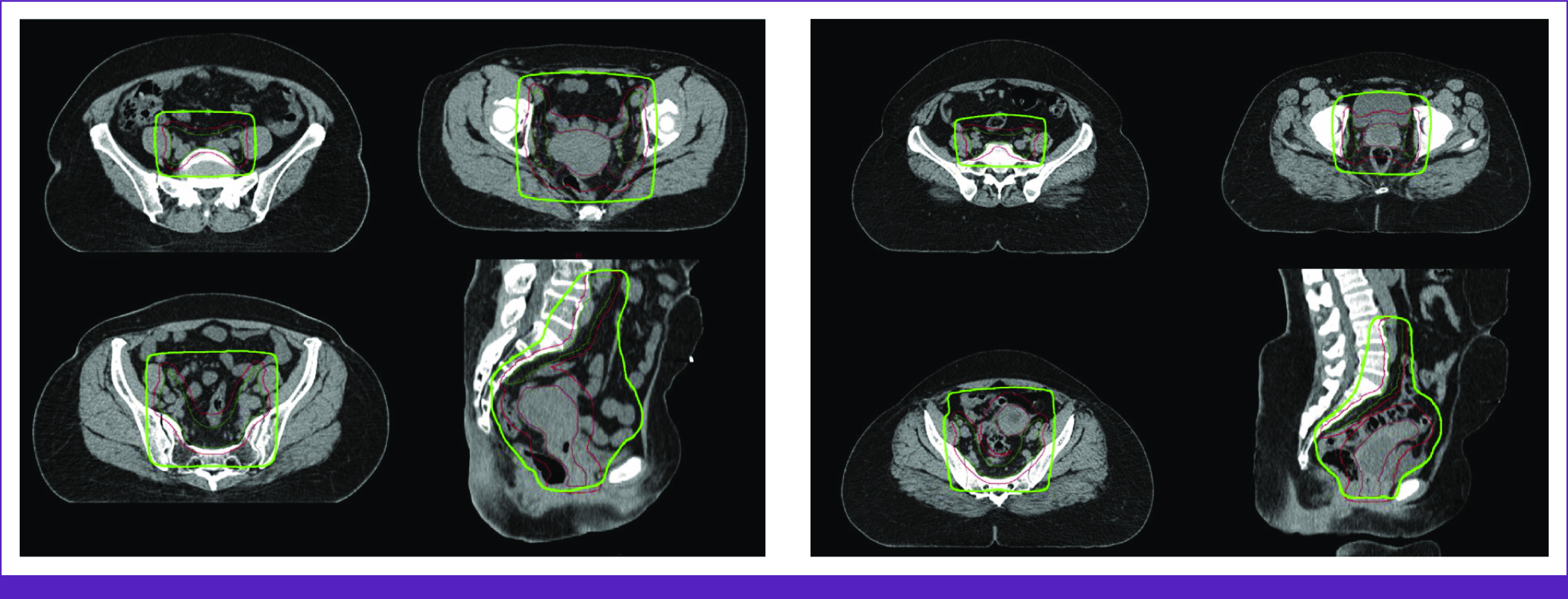
Sample of 3DCRT plans generated by the RPA. The CTV nodal contours are in green and CTV primary contours are in brown. The PTV is in red. The 95% isodose line is highlighted in light green. 3DCRT, 3D conformal radiation therapy; CTV, clinical target volume; PTV, planning target volume; RPA, Radiation Planning Assistant.

RPA target coverage was also excellent (Fig 2), covering the RPA-generated CTVp with median V45 Gy 100% (IQR, 100%-100%) and RPA-generated CTVn with median V45 Gy 100% (IQR, 100%-100%). The RPA-generated PTVp and PTVn were covered to V95% = 99% on the basis of ORCI-defined plan normalization parameters. The hotspot was <110% for all.

**FIG 3:**
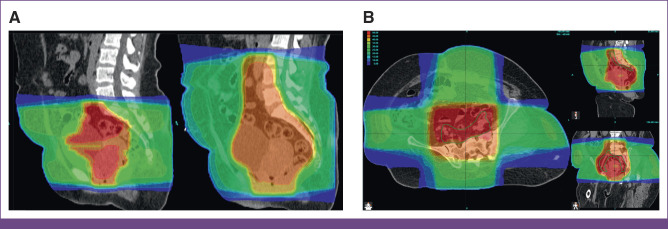
Comparison of 3DCRT plans between ORCI and the RPA. (A) Superior field border extended from L5/S1 on ORCI plan (left) to L3/L4 on RPA plan (right) to ensure the superior edge of the treated field included the aortic bifurcation. (B) RPA plan correctly contoured pelvic lymph node CTV and adjusted treatment field to account for atypical bony anatomy. 3DCRT, 3D conformal radiation therapy; CTV, clinical target volume; ORCI, Ocean Road Cancer Institute; RPA, Radiation Planning Assistant.

The RPA plan was able to adjust for differences in patient anatomy. In one patient where the superior edge of the 3DCRT field would have otherwise been set at L5/S1, the RPA systematically attempted to set the superior edge of the treated field at the aortic bifurcation, which was closer to L3/L4 (Fig 3A). In another patient with left gluteal atrophy and rotated pelvic bones, the RPA was able to correctly contour the CTVn and adjust the treatment field despite the atypical bony anatomy (Fig 3B).

When superimposed on the original ORCI clinical contours, the RPA plan also provided excellent coverage. The coverage of ORCI-CTVp with median V45 Gy was 100% (IQR, 100%-100%) for the RPA plan and the coverage of ORCI-CTVn with median V45 Gy was 99.9% (IQR, 99.6%-100%).

## DISCUSSION

Compared with the median cervical cancer 3DCRT planning timeline in this study of 7 days, we found that the RPA was able to generate high-quality RT contours and plans within minutes. The findings from this study suggest that the RPA has promising potential to improve workflow efficiency and yield high standards of treatment quality in resource-constrained settings, which is a priority for large-volume centers using 3DCRT for cervical cancer.

An expanding body of evidence supports the clinical utility of automation assistance,^7,13,15^ and commercial automation solutions are becoming increasingly available as a result (eg, RadFormation, Limbus Contour, RapidPlan, etc). Similarly, our findings demonstrated the clinical acceptability of automation-assisted contours and plans, although a few novel points arise that may especially apply in resource-limited settings.

RPA contours were successfully generated in the majority of patients (97%) and were approved within 2 minutes of edit time. Although there was high agreement (median DSC scores exceeding 0.70) between clinical and RPA contours for normal structures, such as the rectum and bladder, RPA contours also included bilateral femurs, bowel bag, and bone marrow, which are commonly omitted in ORCI clinical practice because of time constraints. The ease of availability of these automated contours could enable ORCI physicians to access a richer dose-volume data set for plan evaluation. This may allow the radiotherapy team to more easily and reliably develop treatment plans that meet previously unmeasured plan constraints, which would promote optimal treatment outcomes.

Lower contour agreement was noted between the target volumes, CTVp and CTVn, with the RPA contours including more posterolateral margin on vessels, less muscle, more presacral nodes, and additional extent of CTVp inferiorly (Fig 1). Because these contours were drawn in the early years of 3DCRT at ORCI, some of these clinical contour insufficiencies reflect the steep learning curve required to transition from 2D to 3DCRT. It should be noted that contours are not a requirement for 3DCRT, and bony-landmark–based 4-field box radiation planning is also widely practiced, efficient, safe, and acceptable. Yet, to respond to a clinical need, this project inspired further cross-institutional educational exchange to review pelvic anatomy, CT anatomy, and nodal contouring guidelines. This project revealed that collaborative partnerships are integral to successful implementation of new technology, such as contouring, 3DCRT, and the RPA.

RPA plans were consistently approved with <4 minutes of edit time by both reviewers. The RPA system's ability to generate plans on the basis of soft-tissue anatomy poses two distinct advantages. The first is systematic determination of the superior edge of the treatment field. Although the RPA system has the ability to provide bony-landmark–based whole pelvis fields, ORCI physicians were interested in transitioning from bony landmarks to soft-tissue–based landmarks for the superior edge of the standard pelvic field. The RPA was able to accurately and consistently identify the aortic bifurcation as the superior edge in all submitted plans. Second, for patients with atypical or asymmetric pelvic anatomy, the RPA was still able to provide a remarkably accurate estimation of where the elective pelvic lymph node volume ought to be contoured and generated a clinically excellent plan according to this estimation. Future RPA implementation efforts where more patients with atypical or asymmetric anatomy will help illuminate the extent to which RPA can reliably adapt to inevitable variations in human anatomy.

Retrospective chart reviews of treatment initiation timelines support the need for strategies to expedite high-quality and high-throughput radiation services. All patients presented with symptoms significant enough to result in a decline in ECOG PS, which is a strong motivator for early initiation of treatment. The treatment planning step represents only one cause for delay in the overall time from diagnosis to treatment initiation in resource-limited settings (Table 2); however, even small incremental time-savings in treatment planning can become a potentially high-impact intervention when compounded by large patient volumes. The benefit becomes enormous if routine patient-specific workflows are reduced from 7 days by manual means to <1 day with automation assistance. The only barrier to RPA contour and plan generation in our study occurred in one patient where a single slice was missing from the exported simulation CT and this error can be readily circumvented with quality control in future implementation efforts.

This was designed as a pragmatic study to investigate how the RPA could be introduced into a resource-limited setting without specific requirements for how facile the existing medical system is with contouring or 3DCRT treatment planning, or specifications for reliability of wireless internet. A limitation of this approach in contour agreement analysis was nonuniformity in ORCI PTV labeling, summations, and expansions. PTV contours and coverage metrics could not be directly compared in this analysis. Several additional OARs, such as bowel bag, femurs, and bone marrow, could not be compared.

The workflow efficiencies achieved in this study required high-speed and reliable internet access and an on-site research team to troubleshoot upload/download issues throughout the study period. If RPA were adopted for clinical use, these issues may be addressed in the future by training dedicated staff members to incorporate RPA seamlessly into an LMIC clinic's workflow. Although the findings of this study only apply to 3DCRT for patients with cervical cancer, the RPA platform is under development for a variety of other cancer sites that can be tested in a similar manner.

Lastly, the RPA is a tool that requires physician oversight to determine whether it is being applied in the right clinical context, confirm accuracy of contours, and assess safety of radiation plans. As modern RT linear accelerators become increasingly available in sub-Saharan Africa and other resource-constrained settings, collaborative partnerships focused on assisting transitions from 2D to 3DCRT will be critical to ensure that new technologies, such as RPA, are implemented successfully.

In conclusion, this study demonstrated the potential time-savings, workflow efficiency, and clinical acceptability of implementing the RPA system for automation-assisted 3DCRT contouring and planning in a resource-constrained setting. The RPA generated accurate contours and consistently produced high-quality and safe radiation plans that required minimal manual editing before clinical approval. Further efforts to integrate this tool into clinical workflows has high potential to significantly decrease RT planning timelines, increase access to high-quality RT, and critically expand the clinical care capacity of existing radiotherapy centers serving resource-constrained settings.

This study was designed and conducted in close partnership between local clinicians and researchers and an international study team. All research priorities were shaped according to local clinical needs. To protect health information of study subjects, institutional review board approval was sought on a local and national level, with only deidentified information shared with international study partners. Research activities were conducted by a local research team and funded with global-health designated grants. All members of the local research team are represented in the study's authorship line.

## Data Availability

Research data are stored in an institutional repository and will be shared upon request to the corresponding author.

## References

[1] Sung H Ferlay J Siegel RL et al Global cancer statistics 2020: GLOBOCAN estimates of incidence and mortality worldwide for 36 cancers in 185 countries CA Cancer J Clin 71 209 249 2021 33538338 10.3322/caac.21660

[2] Knaul FM Arreola-Ornelas H Rodriguez NM et al Avoidable mortality: The core of the global cancer divide JCO Glob Oncol 4 1 12 2018 10.1200/JGO.17.00190 PMC6223530 30096010

[3] Grover S Xu MJ Yeager A et al A systematic review of radiotherapy capacity in low- and middle-income countries Front Oncol 4 380 2014 25657930 10.3389/fonc.2014.00380 PMC4302829

[4] Grover S Balogun OD Yamoah K et al Corrigendum: “Training global oncologists: Addressing the global cancer control problem” Front Oncol 5 133 2015 26114095 10.3389/fonc.2015.00133 PMC4461853

[5] Atun R Jaffray DA Barton MB et al Expanding global access to radiotherapy Lancet Oncol 16 1153 1186 2015 26419354 10.1016/S1470-2045(15)00222-3

[6] Rodin D Jaffray D Atun R et al The need to expand global access to radiotherapy Lancet Oncol 15 378 380 2014 24694630 10.1016/S1470-2045(14)70121-4

[7] McCarroll RE Beadle BM Balter PA et al Retrospective validation and clinical implementation of automated contouring of organs at risk in the head and neck: A step toward automated radiation treatment planning for low- and middle-income countries JCO Glob Oncol 4 1 11 2018 10.1200/JGO.18.00055 PMC6223488 30110221

[8] Yang J Veeraraghavan H Armato SG et al Autosegmentation for thoracic radiation treatment planning: A grand challenge at AAPM 2017 Med Phys 45 4568 4581 2018 30144101 10.1002/mp.13141 PMC6714977

[9] Rhee DJ Jhingran A Kisling K et al Automated radiation treatment planning for cervical cancer Semin Radiat Oncol 30 340 347 2020 32828389 10.1016/j.semradonc.2020.05.006 PMC7446764

[10] Krayenbuehl J Zamburlini M Ghandour S et al Planning comparison of five automated treatment planning solutions for locally advanced head and neck cancer Radiat Oncol 13 170 2018 30201017 10.1186/s13014-018-1113-z PMC6131745

[11] Olsen LA Robinson CG He GR et al Automated radiation therapy treatment plan workflow using a commercial application programming interface Pract Radiat Oncol 4 358 367 2014 25407855 10.1016/j.prro.2013.11.007

[12] Zhu M Bzdusek K Brink C et al Multi-institutional quantitative evaluation and clinical validation of Smart Probabilistic Image Contouring Engine (SPICE) autosegmentation of target structures and normal tissues on computer tomography images in the head and neck, thorax, liver, and male pelvis areas Int J Radiat Oncol Biol Phys 87 809 816 2013 24138920 10.1016/j.ijrobp.2013.08.007

[13] Kisling K Zhang L Simonds H et al Fully automatic treatment planning for external-beam radiation therapy of locally advanced cervical cancer: A tool for low-resource clinics JCO Glob Oncol 5 1 9 2019 10.1200/JGO.18.00107 PMC6426517 30629457

[14] Olanrewaju A Court LE Zhang L et al Clinical acceptability of automated radiation treatment planning for head and neck cancer using the Radiation Planning Assistant Pract Radiat Oncol 11 177 184 2021 33640315 10.1016/j.prro.2020.12.003 PMC9272530

[15] Lustberg T van Soest J Gooding M et al Clinical evaluation of atlas and deep learning based automatic contouring for lung cancer Radiother Oncol 126 312 317 2018 29208513 10.1016/j.radonc.2017.11.012

[16] Khamis SI Mrema AS Katanga J et al Survival in cervical cancer and its predictors at Ocean Road Cancer Institute from January to December 2012 JCO Glob Oncol 7 734 739 2021 34010012 10.1200/GO.20.00616 PMC8162959

[17] Pecorelli S Revised FIGO staging for carcinoma of the vulva, cervix, and endometrium Int J Gynecol Obstet 105 103 104 2009 10.1016/j.ijgo.2009.02.012 19367689

